# P^3^DB: An Integrated Database for Plant Protein Phosphorylation

**DOI:** 10.3389/fpls.2012.00206

**Published:** 2012-09-07

**Authors:** Qiuming Yao, Curtis Bollinger, Jianjiong Gao, Dong Xu, Jay J. Thelen

**Affiliations:** ^1^Department of Computer Science, University of MissouriColumbia, MO, USA; ^2^Bond Life Science Center, University of MissouriColumbia, MO, USA; ^3^Computational Biology Center, Memorial Sloan-Kettering Cancer CenterNew York, NY, USA; ^4^Department of Biochemistry, University of MissouriColumbia, MO, USA

**Keywords:** protein phosphorylation, P^3^DB, mass spectrometry, plants, data repository, phosphoproteomics

## Abstract

Protein phosphorylation is widely recognized as the most widespread, enzyme-catalyzed post-translational modification in eukaryotes. In particular, plants have appropriated this signaling mechanism as evidenced by the twofold higher frequency of protein kinases within the genome compared to other eukaryotes. While all aspects of plant protein phosphorylation research have grown in the past 10 years; phosphorylation site mapping using high-resolution mass spectrometry has grown exponentially. In *Arabidopsis* alone there are thousands of experimentally determined phosphorylation sites. To archive these events in a user-intuitive format we have developed P^3^DB, the Plant Protein Phosphorylation Database (p3db.org). This database is a repository for plant protein phosphorylation site data, currently hosting information on 32,963 non-redundant sites collated from 23 experimental studies from six plant species. These data can be queried for a protein-of-interest using an integrated BLAST module to query similar sequences with known phosphorylation sites among the multiple plants currently investigated. The paper demonstrates how this resource can help identify functionally conserved phosphorylation sites in plants using a multi-system approach.

## Introduction

Protein phosphorylation is one of the most ubiquitous post-translational modifications (PTM), controlling signaling pathways, metabolic, and cellular processes. It is evident that plants are particularly adept at this form of protein modulation as the genome of the reference plant *Arabidopsis thaliana* contains slightly over 1000 protein kinases, at least twice as many on a gene frequency basis compared to man or fungi (Wang et al., 2007). As most kinases are capable of phosphorylating multiple proteins, it is likely that the majority of the plant proteome has the potential to be phosphorylated, if only transiently. Technology and methodology to experimentally catalog the plant phosphoproteome have existed for over a decade (Neubauer and Mann, 1999; Ficarro et al., 2002; Gruhler et al., 2005), and there has been a steady increase in the number of mapped phosphorylation sites collectively from model and crop plants (Nuhse et al., 2004, 2007; Wolschin and Weckwerth, 2005; Benschop et al., 2007; de la Fuente van Bentem et al., 2008; Sugiyama et al., 2008; Whiteman et al., 2008; Hsu et al., 2009; Ito et al., 2009; Jones et al., 2009; Li et al., 2009; Reiland et al., 2009; Wang et al., 2009; Chen et al., 2010; Grimsrud et al., 2010; Kline et al., 2010; Nakagami et al., 2010; Bi et al., 2011; Engelsberger and Schulze, 2012; Meyer et al., 2012).

The information related to phosphorylation site data is multi-dimensional and includes experiment-specific information (i.e., meta-data, such as experimental parameters and data quality metrics), peptide sequence, phosphorylation site, mass spectra, etc. As a result there is a clear need to hierarchically store and organize the increasingly large phosphoproteomics data, motivating the creation of phosphorylation databases in the past few years. HPRD, the Human Protein Reference Database (Prasad et al., 2009) provides a wide range of data of phosphorylation and other modifications, but the data are limited to human. Phosphosite (Hornbeck et al., 2012) also provides large datasets but mainly on human, rat, and mouse. Phospho.ELM (Diella et al., 2008; Dinkel et al., 2011) also contains data on human and mouse. PhosphoPep (Bodenmiller et al., 2008) archives phosphoproteomic data from yeast, worm, and fly in addition to human. PHOSIDA (Gnad et al., 2011) provides phosphoproteomic data from a comprehensive group of organisms from human to bacteria, but again no plant data are available. UniProt (Farriol-Mathis et al., 2004) is another source of protein sequences and annotations for all kind of modifications; however, phosphorylation data are limited (Table 1). As few of these databases accommodate plant phosphoproteomics data two recent databases were created, PhosPhAt and P^3^DB. PhosPhAt (Heazlewood et al., 2008; Durek et al., 2010) is a database that specifically maintains experimental phosphorylation site data for *Arabidopsis*, whereas the Plant Protein Phosphorylation DataBase, or P^3^DB (Gao et al., 2009), is a comprehensive repository for all plant phosphoproteomic data, including *Arabidopsis*.

**Table 1:** **Phosphorylation site data for plants in UniProt and P^3^DB 2.0**.

	Ploidy	P^3^DB				UniProt (February 2012)			
		Protein	S	T	Y	Protein	S	T	Y
*Arabidopsis thaliana*	2	3930	10,166	2656	801	707	1110	212	25
*Brassica napus*	4	325	484	218	116	0	0	0	0
*Glycine max*	4	1451	2201	397	141	1	1	0	0
*Medicago truncatula*	4	980	2688	550	113	0	0	0	0
*Oryza sativa*	2	4829	10,025	1706	586	0	0	0	0
*Zea mays*	4	86	100	14	1	4	3	1	0
Total for plants		11,601	25,664	5541	1758	712	1114	213	25

*Statistics are calculated from six organisms*.

P^3^DB is both a web portal and an integrated database specifically for archiving and disseminating plant protein phosphoproteomics data. It integrates data from different plants, experimental approaches, and spectral search algorithms. P^3^DB is also a reliable data source for computational analysis since all of its data are downloadable. It can help to easily test a hypothesis and make inferences based on the high-quality phosphoproteomics data. For example, disorder is a feature to measure the local structure stability of proteins. The higher the disorder score, the more flexible that local structure is likely to be. There are hypotheses that protein phosphorylation occurs preferentially within disordered regions (Iakoucheva et al., 2004). P^3^DB provides a resource to further explore this relationship.

Since the release of P^3^DB 1.0 (Gao et al., 2009), this database has been under extensive development in terms of software/tool infrastructure, phosphoproteome coverage, and integration of these datasets with contextual information about phosphorylation site assignment and function. In this paper, we highlight the current status of the database and some of the recent developments since the original publication of the database (Gao et al., 2009). We also further explore the relationship between protein phosphorylation and disorder analysis using this rich source of plant phosphoproteomics data.

## Database Infrastructure

### Data

In P^3^DB version 2.0, phosphoproteomics data were integrated from 23 experimental studies including 11,601 phosphoproteins harboring 32,962 non-redundant phosphorylation sites. This new dataset covered six different organisms (*A. thaliana*, *Brassica napus*, *Glycine max*, *Medicago truncatula*, *Oryza sativa*, and *Zea mays*), with different experimental designs including mass spectrometry instrumentation (e.g., chromatography method, dissociation techniques, etc.) as well as data mining algorithms (and associated output). Consequently, the data were of different quality. To maintain the high-quality data within P^3^DB, while also expanding this database in accordance with the growing body of global phosphoproteomic studies, simple quality control standards were implemented. To be deposited into P^3^DB, datasets must meet the following criteria: FDR <1%; precursor mass accuracy <15 ppm; and PTM-score >84, in which case identification of the phosphorylation site is statistically significant compared to background. Additionally, all database searching must be performed with homologous proteome databases.

### Interface

P^3^DB 2.0 has a new user-friendly web interface with better visualization for phosphoproteomics data and for data searching. Hierarchical design was applied to the front-end display as well as the database schema which not only facilitates user access to the interested portion of the database but also provides a comprehensive viewer to explore the data from all aspects. The data were first classified by organisms as the highest level, then proteins, followed by peptides, finishing with phosphorylation sites. Annotated mass spectra were mapped to the peptides if provided from experimental side. The data sources were listed in a drop-down list, which can be used as a filter by user selection at any scale level. Phosphoproteins in P^3^DB are also seamlessly linked to prediction results from the phosphorylation site prediction tool Musite (Gao et al., 2010). More features can be seen directly from the example section.

### Standard output

Besides just exploring the details and information related to site phosphorylation, P^3^DB also allows the user to extract the batch data from the download module. Phosphoprotein report, phosphosite report, non-redundant phosphopeptide report, and redundant phosphopeptide report are four of our standardized file formats. Phosphoprotein reports contain all the protein sequences and phosphosite reports have all phosphorylated sites listed. Therefore, by simply combining these two report files, it is easy to convert to MusiteXML, which can be uploaded to Musite for additional analysis or prediction.

### Implementation

MySQL was used for storage, maintenance, and operation of P^3^DB. The front-end web interface was implemented in PHP, which is a popular scripting language for dynamic webpages. In P^3^DB 2.0, a well-defined and packaged JavaScript called jQuery was used to enhance the interface of the website and improve user experience. The database, specifically the display of data, was both designed as a hierarchical structure supporting navigation among proteins, peptides, phosphorylation sites, and mass spectra (if available). Original citations were also linked to all of the archived data.

## Example of Use

### Data analysis

**Figure 1:**
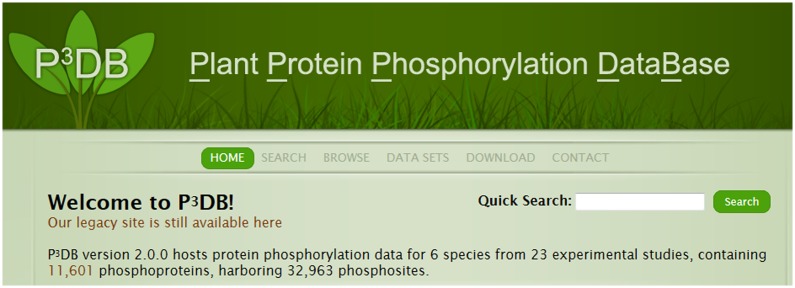
**Main page of P3DB 2.0 website**.

Through the portal http://p3db.org, the new home page for the P^3^DB 2.0 website is shown (Figure 1). Table 1 lists the fraction of phosphorylated Ser, Thr, and Tyr in each organism and the comparison to the UniProt release (February 2012).

**Figure 2:**
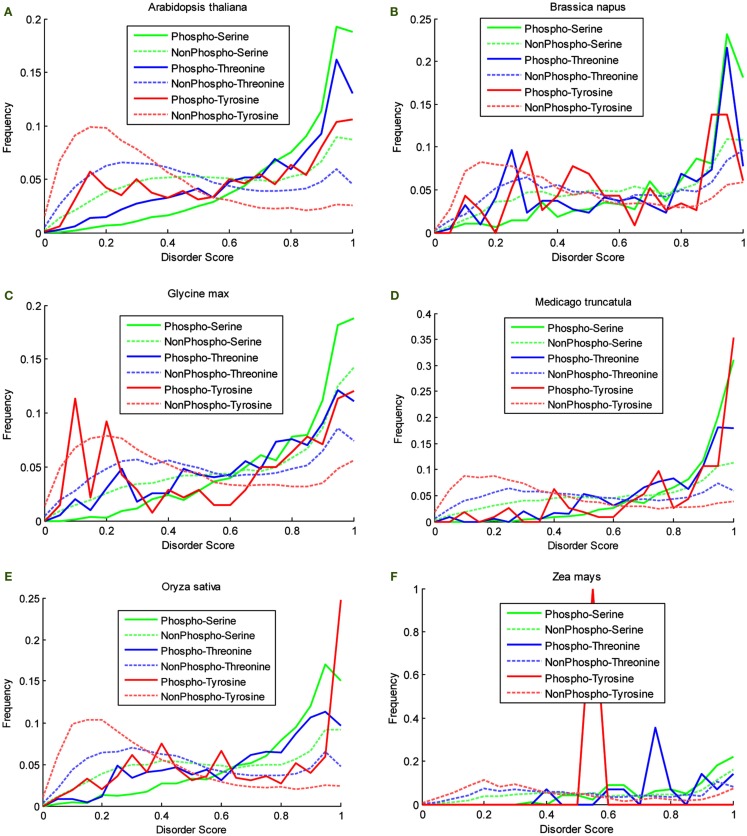
**Disorder score distribution in six organisms**. **(A)** *Arabidopsis thaliana*, **(B)** *Brassica napus*, **(C)** *Glycine max*, **(D)** *Medicago truncatula*, **(E)** *Oryza sativa*, and **(F)** *Zea mays*.

P^3^DB 2.0 provides a great source to analyze the disorder characteristic that could be potentially related to site phosphorylation. Disorder score for each phosphosite can be calculated by VSL2B (Obradovic et al., 2005) ranging from 0 to 1. All of the scores were classified into the 200 equally divided bins. Then the obtained histograms were normalized by the total number of counted sites and allowing the empirical distribution to be obtained. For each organism in P^3^DB 2.0, the empirical distributions on both phosphorylated and non-phosphorylated sites were plotted (Figure 2). The horizontal axis from left to right shows the local structure from non-disordered/low-disordered to high-disordered region. The normalized frequency in the vertical axis depicts the density of the empirical distribution.

Two-sample Komolgorov–Smirnov test was used to determine if the two disorder distributions were significantly different for phospho- and non-phosphosites. The results are listed in Table 2.

**Table 2:** **Results of Komolgorov–Smirnov test for disorder distributions of phospho- and non-phosphosites**.

	K–S statistics			*p*-Value		
	S	T	Y	S	T	Y
*Arabidopsis thaliana*	0.4286	0.4286	0.3810	0.0290	0.0290	0.0706
*Brassica napus*	0.4762	0.4762	0.3333	0.0108	0.0108	0.1545
*Glycine max*	0.2857	0.2381	0.4762	0.3038	0.5309	0.0108
*Medicago truncatula*	0.4286	0.4286	0.5238	0.0290	0.0290	0.0036
*Oryza sativa*	0.3810	0.2381	0.2381	0.0706	0.5309	0.5309
*Zea mays*	0.3810	0.6190	0.9524	0.0706	0.0003	0.0002
Total for plants	0.3810	0.4286	0.3810	0.0706	0.0290	0.0706

### Specific protein study

Besides the meta-data analysis, P^3^DB provides multiple tools for searching, retrieving, and visualizing phosphoproteomics data. In the following example we show the process of retrieving the homologous proteins and exploring the potential conservative sites among different studies within the P^3^DB.

**Figure 3:**
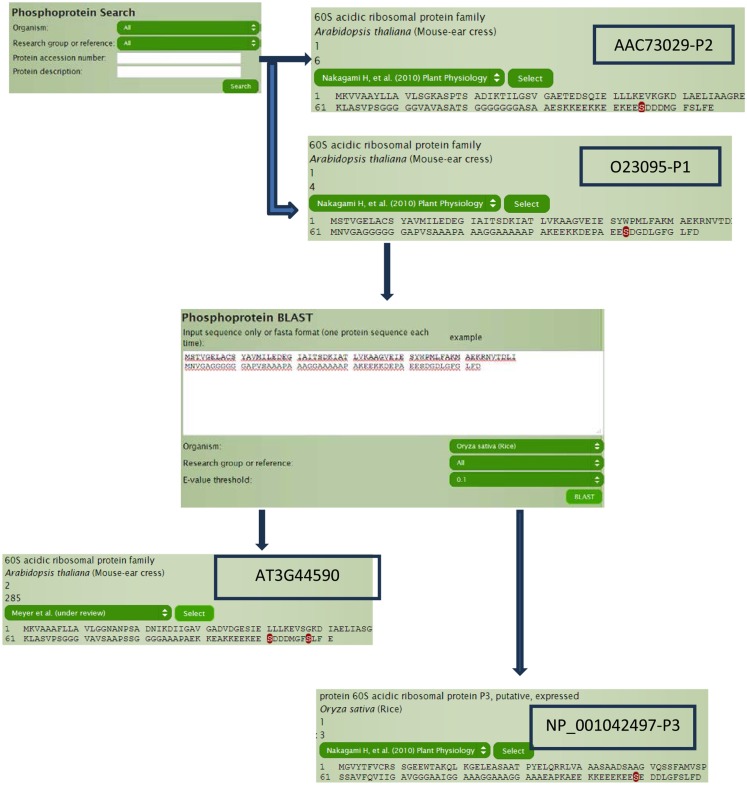
**Example of using search engine to access the homologous proteins**.

60S acidic ribosomal protein plays a very important role in the elongation step of protein biosynthesis, which exists as heteromeric complex of subunits P1, P2, and P3 (Tchorzewski, 2002). The *Arabidopsis* homologs (Figure 3), P1 subunit O23095 (AT4G00810) and P2 subunit AAC73029 (AT2G27710; Tchorzewski, 2002), can be obtained directly by typing O23095 and AAC73029 in the search box without even knowing the TAIR numbers, because the current P^3^DB 2.0 supports more than 25 types of different accession number (IDs) in the search module and does the mapping internally. Other than querying by accession number, data can also be searched using protein description, annotations, and protein names within the search module. The search module also allows the user to filter by organisms or studies to narrow down the search space. On the protein page, all accession numbers in the system can be obtained so that we can easily know or verify the TAIR numbers (AT4G00810 and AT2G27710) for our target proteins O23095 and AAC73029. The protein page shows the phosphorylation sites on the whole sequence with the sites highlighted. By looking at the protein pages for our P1 and P2 subunits (Figure 3), it is obvious that the location of the only phospho-serine is conserved in both proteins, with a motif EES(*)DDD.

To determine if this phospho-serine residue is conserved, we can perform a BLAST search through P^3^DB using the protein sequence O23095 and find the orthologous protein in rice (*Oryza sativa*). Although the best hit is a P3 subunit, it does contain the conserved phosphorylation site too. We can also try to search for the other homologous proteins within *Arabidopsis*, i.e., paralogs. With the best hit of AT3G44590, two sites are observed to be phosphorylated other than the one that is strongly conserved. Sequence conservation of phosphorylation sites can be a useful indicator for conservation of regulatory function.

**Figure 4:**
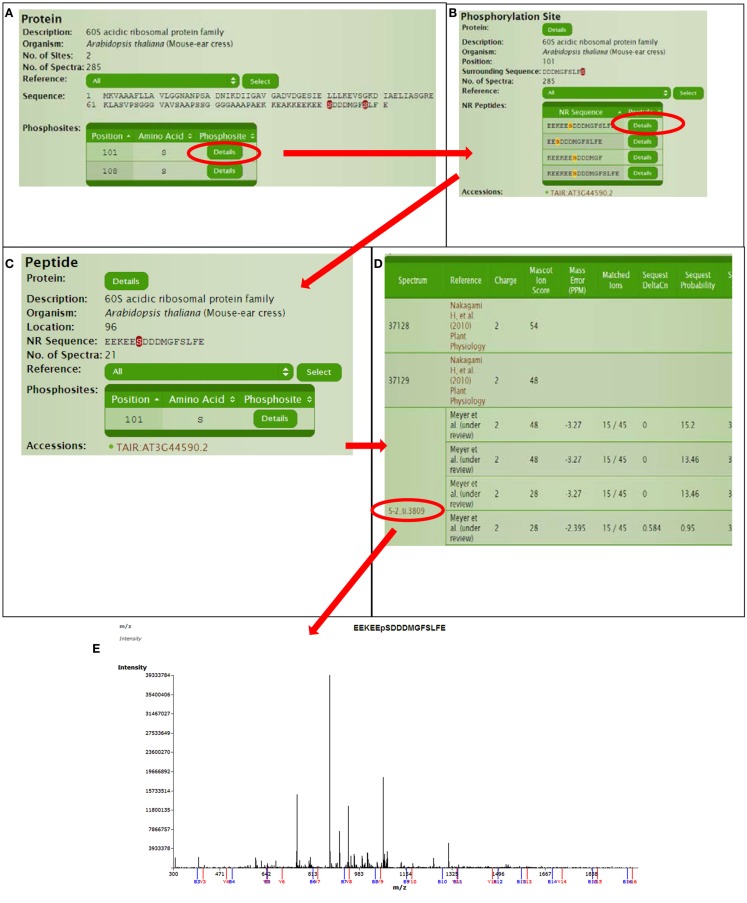
**Detailed information for a certain phosphorylation site or peptide**. **(A)** Protein page, **(B)** Phospho-site page, **(C)** Peptide page, **(D)** Spectra parameters on peptide page, **(E)** Spectra viewer.

Continuing with protein AT3G44590, we can explore for further information in a hierarchical manner (Figure 4). The protein page usually has multiple links for the phosphorylated sites where site details are shown like flanking sequence and the number of available spectra in P^3^DB. Since the same site might be observed in different peptides, each non-redundant peptide link leads to the specific peptide page. The peptide page contains the details of the experimental condition and parameters, each of which is mapped to the spectrum. If the annotated mass spectrum is available (we request this from all users who deposit data into P^3^DB), the visualization tool will bring the mass spectrum and annotated peptide fragmentation series in the new screen.

Furthermore, the long green button “send to Musite for phosphorylation prediction” on the protein page will send the sequence to Musite on the fly to do prediction. Therefore, the standard output of P^3^DB facilitates the communication between the experimental reliable dataset with the more extensive computational tools and analyses.

## Discussion

Table 1 shows that P^3^DB 2.0 contains considerably more plant phosphoproteomics data than the recent version (February 2012) of the comprehensive protein database UniProt. This is because the phosphorylation data within UniProt is almost exclusively from *Arabidopsis*. P^3^DB 2.0 has a wide range of different plant species, reflecting the expansion of phosphoproteomics research in Viridiplantae. The overall variability in phospho-amino acid frequency among the various plants likely reflects the preliminary nature of cataloging the plant phosphoproteome, particularly for crop species.

The plots in Figure 2 show the different disorder distribution for phospho- and non-phosphosites among different organisms. The quantitative analysis by Komolgorov–Smirnov test (Table 2) shows that in most of the cases the disorder scores for each amino acid were differentially distributed, and for phospho- and non-phosphosites the disorder scores were also significantly different.

Although the plots are more descriptive (Figure 2), they are comparable since they are calculated on a fixed scale. For the non-phosphorylated sites, the density curves are almost the same among six species. For the phosphorylated sites, since the data are much sparser, the density curves are not as smooth as the non-phosphorylated curves, and they also differ from each other. However, the relative relationship among these density curves is preserved among different species. First, the disorder scores of the non-phosphorylated sites tend to be distributed in the lower disordered regions more so than the phosphorylated sites. Especially for the curves of non-phosphorylated Thr and Tyr, the maxima, and the large population are in the regions of less than 0.5, while for the phosphorylated sites, the density curves almost grow exponentially in the highly disordered region. Although for Ser, both curves grow in the highly disordered region, it is noteworthy that the non-phosphorylated density is above the phosphorylated density in the lowly disordered region, and this relationship is inverted in the highly disordered region. This clearly shows that a large population of the phosphorylated sites has the tendency to be in the disordered region while the non-phosphorylated sites are more likely to be located in non-disordered regions for all the species in this study. Second, the distribution patterns for Ser, Thr, and Tyr differ; however, the relationship in both highly disordered and lowly disordered regions is the same in different species. In the non-phosphorylated case, the Tyr curve is above the Thr, and the Ser curve is at the bottom, while in the phosphorylated case, the relation flips as the order of high density to low is Ser, Thr, and Tyr.

P^3^DB 2.0 displays the data in a relational, hierarchical manner that integrates proteins, peptides, phosphosites, and spectra for each phosphorylation event. Various search and query tools (search by protein accession, description, annotations, protein names, and protein/peptide sequences) are embedded within the P^3^DB website framework allowing for seamless interrogation of the data without leaving the site. For example, the search module provides the user with multiple ways to access to proteins of interest and the BLAST search tool allows for comparative studies at the primary sequence level.

In summary, P^3^DB version 2.0 now has become an integrated data bank and portal driven by the rapidly growing field of high-throughput phosphorylation site mapping. For bioinformaticist or experimental biologist, P^3^DB provides both the tools and experimental resources for querying plant protein phosphorylation data.

## Conflict of Interest Statement

The authors declare that the research was conducted in the absence of any commercial or financial relationships that could be construed as a potential conflict of interest.
